# Insulin resistance is associated with an unfavorable outcome among non-diabetic patients with isolated moderate-to-severe traumatic brain injury – A propensity score-matched study

**DOI:** 10.3389/fneur.2022.949091

**Published:** 2022-07-28

**Authors:** Cheng Cao, Huxu Wang, Heng Gao, Wei Wu

**Affiliations:** Department of Neurocritical Intensive Care Unit, Jiangyin Hospital Affiliated to Nantong University, Jiangyin, China

**Keywords:** traumatic brain injury, insulin resistance, HOMA2, hyperglycemia, glycemic variability, prognosis

## Abstract

**Background:**

Hyperglycemia is an independent risk factor for the poor prognosis in patients with traumatic brain injury (TBI), and stress-induced impaired insulin function is the major factor of hyperglycemia in non-diabetic patients with TBI. Several types of research suggested that insulin resistance (IR) is related to the poor prognosis of neurocritical ill patients; here we focused on the role of IR in non-diabetic patients after TBI.

**Methods:**

We performed a prospective observational study with the approval of the Ethics Committee of our institute. IR was accessed *via* the update Homeostasis Model Assessment (HOMA2) of IR, a computer-calculated index by glucose and insulin level. HOMA2 ≥ 1.4 was considered as the threshold of IR according to the previous studies. The glycemic variability (GV) indices were calculated by fingertip blood glucose concentration at an interval of 2 h within 24 h to explore the relationship between IR and GV. The outcome was the 6-month neurological outcome evaluated with the Glasgow outcome scale.

**Results:**

A total of 85 patients with isolated moderate-to-severe TBI (admission GCS ≤ 12) were finally included in our study, 34 (40%) were diagnosed with IR with HOMA2 ≥ 1.4. After propensity score matching (PSM), 22 patients in IR group were matched to 34 patients in non-IR group. Patients with IR suffered increased systemic glycemic variation after isolated moderate-to-severe TBI. IR was a significant factor for the poor prognosis after TBI (*OR* = 3.25, 95% *CI* 1.03–10.31, *p* = 0.041).

**Conclusions:**

The IR estimated by HOMA2 was associated with greater GV and an unfavorable outcome after isolated moderate-to-severe TBI. Ameliorating impaired insulin sensitivity may be a potential therapeutic strategy for the management of TBI patients.

## Introduction

As suggested by the study of the International Mission for Prognosis and Clinical Trial design in TBI (IMPACT), hyperglycemia is widely recognized as a significant prognostic factor of poor prognosis after brain injuries (1). The previous studies further confirmed that glycemic normalization by insulin therapy significantly improves the prognosis of patients with traumatic brain injury (TBI), although there is still controversy over intensive and conventional insulin therapy (2, 3).

Diabetes is one of the most important factors of hyperglycemia and leads to poor prognosis after TBI (4). However, hyperglycemia is also common in non-diabetic TBI patients and is suggested to have a greater impact on poor prognosis with over 50–60% increased mortality rate (5, 6). Moreover, the adverse effect of hyperglycemia in non-diabetic patients is more significant than that in diabetic patients after TBI (6).

Stress-induced impaired insulin function is one of the major factors of hyperglycemia in TBI patients besides diabetes (7). Insulin and insulin signaling have been found to play important roles in both peripheral and central functions (8, 9). Impaired insulin sensitivity may lead to greater glycemic variation and metabolic dysfunction, which was significantly associated with poorer functional outcome in neurocritical ill patients (10–12). Insulin resistance (IR) is also suggested to be associated with a variety of neurological disorders by its effect on the central nervous system (CNS) (13–15).

Several types of research suggested that IR is related to the poor prognosis of neurocritical ill patients (16, 17). Mowery et al. reported IR is still associated with poor prognosis despite tight glucose control in critically ill patients (18). However, there is no study focused on the role of IR in non-diabetic patients after TBI. In this single-center prospective observational study, we explored the incidence of IR and whether greater glycemic variability and poor prognosis after isolated moderate-to-severe TBI are related to IR in non-diabetic patients. We hypothesized IR was associated with increased systemic glycemic variation and would be a significant factor of poor prognosis among non-diabetic patients with TBI.

## Methods

### Study population and data collection

**Figure 1:**
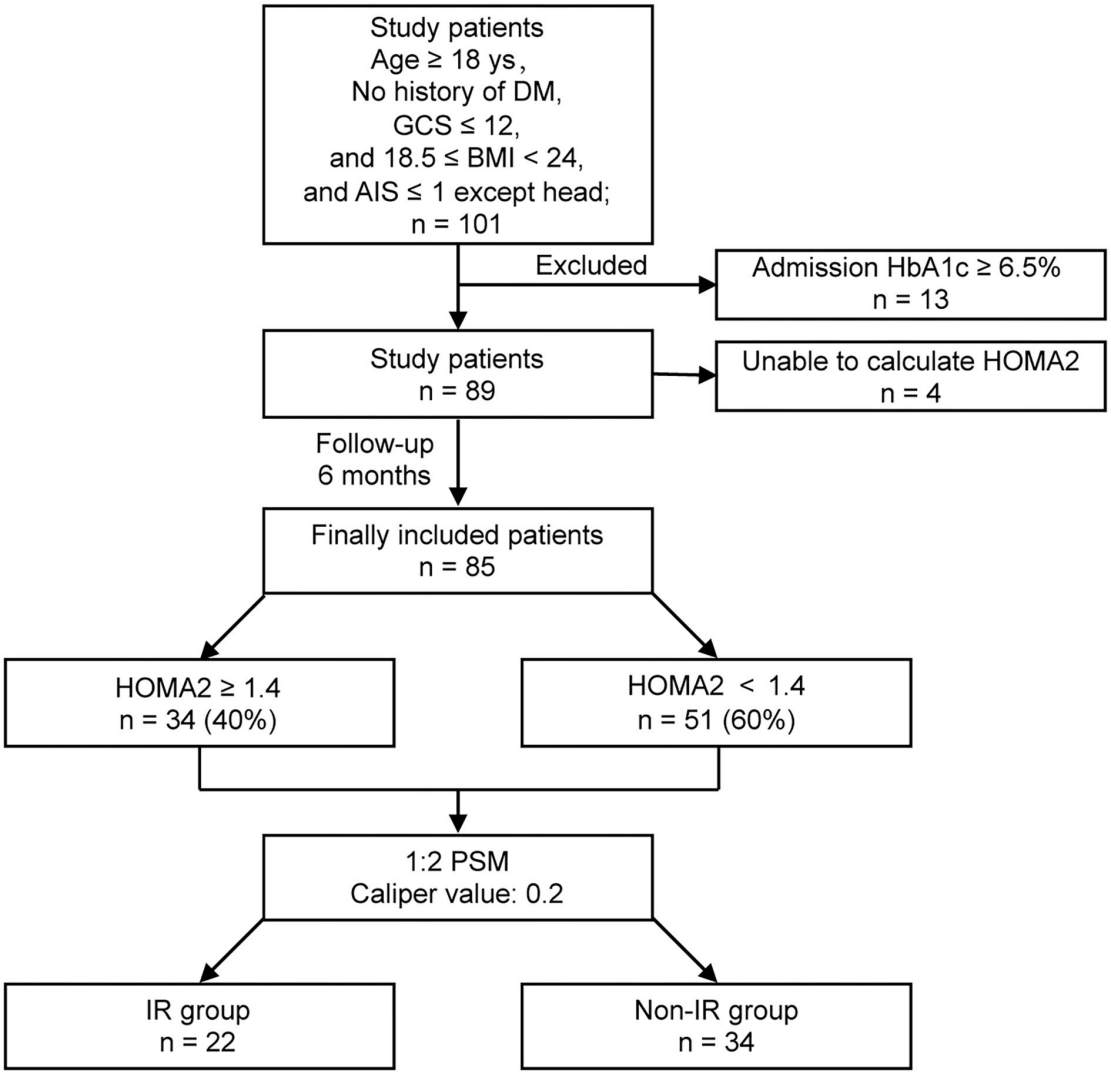
Flow chart of the study groups.

This was a prospective pilot observational study to explore the incidence of IR and whether IR was associated with increased systemic glycemic variation and poor prognosis in non-diabetic patients with isolated moderate-to-severe TBI (Figure 1). Approval was obtained from the Ethics Committee of Jiangyin Hospital Affiliated to Nantong University before initiation of the study (No. 2016012).

Patients who met the following criteria were consecutively recruited to our study: (1) patients with admission Glasgow Outcome Scale (GCS) score ≤ 12; (2) patients with no history of diabetes; (3) patients with normal BMI, 18.5 ≤ BMI < 24; (4) patients with isolated TBI, Abbreviated Injury Scale score ≤ 1 except the head. Candidates with severe chronic diseases or malignant tumors, under the age of 18, and with admission HbA1c ≥ 6.5% were excluded. Informed consent was signed by close relatives of all enrolled patients. Body Mass Index (BMI) was calculated according to the patient's height and weight before injury. The level of glycated hemoglobin (measured by HbA1c) was detected at admission, and further analyzed between groups.

For each study participant, fasting blood glucose (FBG) and fasting insulin (FIns) were collected in the first 24 h after admission. FBG and Fins were used to estimate IR *via* Homeostasis Model Assessment (19) (HOMA, University of Oxford, Oxon, UK). In our study, the updated HOMA, HOMA2 (HOMA2 Calculator software version 2.2.3) was used to estimate IR of the participants, and the calculator is available at: https://www.dtu.ox.ac.uk. The accepted input range for HOMA2 Calculator was shown as below: plasma glucose 3.5–25.0 mmol/L and plasma insulin 20–400 pmol/L. Candidates with unaccepted FBG and FIns values for HOMA2 Calculator were also excluded. According to previous studies, IR was defined as HOMA2 ≥ 1.4 (20).

During the first 24 h after admission, the fingertip blood glucose concentrations were recorded every 2 h for the calculation of glycemic variability (GV) Indices, mean fingertip blood glucose (FtBG), blood glucose standard deviation (SD), coefficient of variation (CV), and mean amplitude of glucose excursions (MAGE) (21, 22). MAGE is one of the most preferred indices in the quantitative evaluation of the short-term within-day GV (23). The GV Indices were calculated using EasyGV (©) software (available free for non-commercial use at www.easygv.co.uk) (24). CV was calculated by dividing SD by the corresponding mean FtBG. The calculating formula of MAGE is shown below:

(1) $$\begin{array}{r} MAGE=\Sigma \;\frac{\lambda }{x},\;if\;\lambda >\nu , \end{array}$$

λ = glucose changes from peak to nadir, *x* = number of valid observations, and *v* = 1 SD of mean glucose for a 24-h period.

Other data regarding sex, GCS, causes of injury, and the IMPACT core variables (age, GCS-Motor score, and pupillary reactivity) (1) were also collected at admission. The causes of injury include road traffic accident, ground level fall, fall from height, and others (e.g., violence and the bruise injury caused by heavy object). The worst Marshall CT score (25) in 24 h after admission was also collected. The GCS score at admission was used to classify the severity of TBI, and GCS-M score was used in the final statistical analysis.

All patients of our study were admitted to 18-bed neurological intensive care unit (NICU). Treatments for study patients were under the direction of evidence-based protocols and management guidelines, such as ICP control strategy, ventilator management, sedation management, indications of surgical operations, nutritional support, stress ulcer prophylaxis, deep venous thrombosis prophylaxis, and antibiotic prophylaxis and therapy (26). In our study, we conducted conventional glycemic targets aimed at keeping glucose levels range of 6–10 mmol/L.

### Study follow-up

The outcome was the 6-month neurological outcome evaluated with the Glasgow outcome scale (GOS): (1) death; (2) persistent vegetative state, severe damage with a prolonged state of unresponsiveness, and a lack of higher mental functions; (3) severe disability, severe injury with a permanent need for help with daily living; (4) moderate disability, no need for assistance in everyday life, employment is possible but may require special equipment; (5) low disability, light damage with minor neurological and psychological deficits. GOS 4–5 is defined as a favorable prognosis, while GOS 1–3 as an unfavorable prognosis.

### Statistical analysis

Normally distributed continuous variables were reported by mean and SD, and compared by Student's *t* test. Non-normally distributed continuous variables were presented by the median and interquartile ranges (IQR), and compared using the Mann–Whitney *U* test. The analyzed categorical variables between groups were conducted by the chi-squared test. A propensity score matching (PSM) procedure was used to balance the co-variable factors of the IR and non-IR groups. SPSS version 26.0 (IBM Corporation, Armonk, NY, USA) was used to perform the statistical analyzes at α = 0.05. The PSM procedure was conducted by SPSS with R 3.5 Plug-in for Statistics.

## Results

### The incidence of IR after TBI

A total of 101 consecutive patients with isolated moderate-to-severe TBI (admission GCS ≤ 12) who had no history of diabetes were admitted to our 18-bed NICU. A total of 13 patients were excluded for their admission HbA1c ≥ 6.5%. A total of 4 patients were excluded for the unqualified glucose or insulin level to calculate HOMA2. Finally, 85 patients were completely investigated. Among the included patients, 34 (40%) were diagnosed with IR with HOMA2 ≥ 1.4, while 51 (60%) were with HOMA2 < 1.4 after TBI (Figure 1).

### Baseline characteristics and PSM analysis

The analysis of characteristics suggested statistically significant differences in gender, FBG level, GCS-Motor score, and pupillary reactivity between the IR and non-IR groups. There were no differences in age, the level of HbA1c, causes of injury and Marshall CT score between the two groups. The nearest neighbor PSM was performed in a 1:2 ratio for gender, FBG level, GCS-Motor score, and pupillary reactivity, with a caliper value of 0.2 for exact matching. After PSM analysis for the co-variables, 22 patients in IR group were matched to 34 patients in non-IR group. All baseline characteristics were well balanced between the two groups in the post-PSM analysis (Table 1).

**Table 1:** Baseline data comparison between the two groups before and after propensity score matching (PSM).

	**Pre-PSM**			**Post-PSM**		
	**HOMA2 < 1.4 (*N* = 51)**	**HOMA2 ≥ 1.4 (*N* = 34)**	***P*-value**	**HOMA2 < 1.4 (*N* = 34)**	**HOMA2 ≥ 1.4 (*N* = 22)**	***P*-value**
**Sex**			0.02^†^			0.51^†^
*Male*	44	22		28	16	
*Female*	7	12		6	6	
**Age (SD)**	53.63 (14.70)	50.53 (12.91)	0.32^×^	53.35 (14.92)	49.32 (12.67)	0.30^×^
**GCS-M (IQR)**	5 (4, 5)	4 (2.75, 5)	0.05^#^	5 (4, 5)	4 (3, 5)	0.27^#^
**HbA1c (SD)**	5.85 (0.42)	5.83 (0.37)	0.30^×^	5.89 (0.42)	5.86 (0.38)	0.77^×^
**Causes of injury**			0.25^*^			0.17^*^
*Road traffic accident*	29	18		18	10	
*Ground level fall*	11	10		6	8	
*Fall from height*	10	3		9	2	
*Others*	1	3		1	2	
**Pupillary reactivity**			0.00^*^			0.34^*^
*Both pupils reacted*	40	20		22	12	
*One pupil reacted*	4	7		2	4	
*No pupil reacted*	7	7		5	4	
**Marshall CT score**			0.20^*^			0.10^*^
*I/II*	19	7		10	4	
*III/IV*	6	2		1	2	
*V*	24	22		18	11	
*VI*	2	3		0	3	
**FBG (IQR)**	6.64 (5.42, 7.50)	8.32 (7.25, 11.38)	0.00^#^	7.25 (6.58, 8.16)	7.38 (6.82, 8.75)	0.40^#^

† Fisher's exact test.

* Pearson's chi-squared test.

# Mann–Whitney U test.

× Student's T test.

### Patients with IR suffered increased systemic glycemic variation after isolated moderate-to-severe TBI

Before PSM, mean FtBG, SD, and MAGE in patients with HOMA2 ≥ 1.4 was higher than that in patients with HOMA2 < 1.4, while no statistically significant association was found between IR and CV. In the analysis between the propensity-matched groups, higher MAGE was still associated with IR after TBI (*p* = 0.031) (Table 2).

**Table 2:** The comparison of Glycemic Variability Indices between the two groups before and after PSM.

**GV indices**	**Pre- PSM**			**Post- PSM**		
	**HOMA2 < 1.4 (*N =* 51)**	**HOMA2 ≥ 1.4 (*N =* 34)**	***P-*value**	**HOMA2 < 1.4 (*N =* 34)**	**HOMA2 ≥ 1.4 (*N =* 22)**	***P-*value**
**Mean FtBG**	7.42	8.66	0.000	8.19	8.32	0.35
(median, IQR)	(6.33, 8.38)	(8.16,11.56)		(7.34, 8.62)	(7.86,8.97)	
**SD**	1.80	2.75	0.001	1.96	2.45	0.21
(median, IQR)	(1.40, 2.34)	(1.76, 3.21)		(1.49, 2.47)	(1.61, 2.97)	
**CV**	0.24	0.26	0.15	0.25	0.25	0.62
(median, IQR)	(0.21, 0.29)	(0.22, 0.34)		(0.22, 0.29)	(0.22, 0.35)	
**MAGE**	3.10	4.68	0.000	3.25	4.53	0.031
(median, IQR)	(2.28, 4.20)	(3.13, 5.35)		(2.38, 4.20)	(2.59, 5.20)	

FtBG, fingertip blood glucose, SD, standard deviation, CV, coefficient of variation, MAGE, mean amplitude of glucose excursions. Tested by Mann–Whitney U test.

### Patients with IR suffered poor 6-month prognosis after isolated moderate-to-severe TBI

The poor prognosis after TBI was significantly more frequent in the IR group as compared to the non-IR group (50.00 *vs*. 23.53%, *p* = 0.041) in the propensity-matched groups. IR was a significant factor for the poor prognosis of non-diabetic patients with isolated moderate-to-severe TBI (*OR* = 3.25, 95% *CI* 1.03–10.31) (Table 3).

**Table 3:** Comparative analysis of Glasgow outcome scale (GOS) between the two groups before and after PSM.

		**HOMA2 < 1.4**	**HOMA2 ≥ 1.4**	***P*-value**
**Pre- PSM**	Unfavorable	11	19	0.001
	Favorable	40	15	
**Post- PSM**	Unfavorable	8	11	0.041
	Favorable	26	11	

## Discussion

We enrolled 85 non-diabetic patients with isolated moderate-to-severe TBI in this 1-year case cohort. Approximately 40% (34/85) of the cohort patients suffered IR with HOMA2 ≥ 1.4. A PSM procedure was used to balance the co-variable factors of the IR and non-IR groups, and the further analysis suggested that IR was associated with increased systemic glycemic variation and a poor prognosis after isolated moderate-to-severe TBI among non-diabetic patients.

Insulin is one of the main treatments for ICU clinicians to treat hyperglycemia of critically ill patients, which is partly similar to the treatment of diabetes. The evaluation of IR is one of the important procedures in the diagnosis and treatment of diabetes. However, the concern of IR is always lacking in non-diabetic hyperglycemia. Clinicians are always confused about the assessments of insulin sensitivity. It is the main reason that restricts ICU clinicians to evaluate insulin sensitivity in non-diabetic patients (27). Though several methods have been used to evaluate the insulin sensitivity, there are still no standardized methods and reference values (27). Hyperinsulinemic-euglycemic clamp (HIEC) is referred to as the gold standard for insulin sensitivity assessment, but the complex procedure limits its use in critically ill patients (28). Indirect indices of IR in fasting state are another insulin sensitivity parameter. HOMA is one of the representative indices (19). With the advantages of simple, economy and almost harmless to patients, HOMA are widely applied in epidemiological investigation, large-scale clinical trials, clinical investigation (29, 30). Several studies have verified the good correlation between estimates of IR derived from HOMA and HIEC (19, 31, 32).

In our study, estimates of insulin sensitivity were assessed by HOMA2 Calculator. IR was defined as HOMA2 ≥ 1.4. Mowery et al. explore IR among the ventilated, critically ill surgical patients with TBI through an adapting multiplier, which is based on the hypoglycemic efficiency of insulin (16). However, the adapting multiplier is a retrospective value, and nutritional support interfered with the dosage of insulin during the insulin therapy procedure, which limited its use. Recently, several researchers used triglyceride-glucose index (TyG) as a simple insulin sensitivity parameter (33). TyG was reported to have a high correlation with HOMA (34, 35). TyG was also suggested to be superior to HOMA in several IR-related diseases (36–38), there was still a study showing that HOMA has advantages yet (39). Considering that IR in acute critical illness may not immediately respond to changes in TyG, it seems more reasonable for TyG to study IR in chronic diseases. It is worthy to study the association of TyG and chronic IR after TBI.

The incidence of IR in our cohort was about 40%, which was significantly higher than the incidence of hyperglycemia in our study (fasting blood glucose ≥ 11.1 mmol/L) and that reported in several studies on TBI (5, 40, 41). Although there are no relevant research reports, it is rational that part of TBI patients with IR may only presented abnormal fasting blood glucose. In our study, only 23.53% patients with HOMA2 ≥ 1.4 were with fasting blood glucose higher than 11.1 mmol/L, and 88.24% ones were with fasting blood glucose higher than 6.1 mmol/L (data do not show). However, this was only the result of a small sample analysis with HOMA2. It is necessary to expand the variety of insulin sensitivity methods and the research sample to reach a comprehensive perspective on the incidence of impaired insulin sensitivity after TBI.

In our study, after balancing the co-variables, our data suggested that poor prognosis after TBI was significantly more frequent in the IR group as compared to the non-IR group (50.00 *vs*. 23.53%, *p* = 0.041). There are other studies reporting IR lower the likelihood of neurological improvement in neurocritical illness both laboratory and clinic. Xin et al. (42) induced IR in an animal model with severe TBI and found that IR was associated with higher modified neurological severity scores after severe TBI. A meta-analysis on acute cerebral ischemia suggested that a higher HOMA index was associated with a higher risk of neurological deterioration (17). Mowery et al. reported that IR indexed by an adapting multiplier was associated with in-hospital mortality after TBI (16).

Insulin resistance can contribute to the poor prognosis of TBI in many ways. Patients with IR presented increased systemic glycemic variation (higher MAGE). An observational study included 5,567 critical ill patients; suggested increased glycemic variability was independently associated with increased mortality among patients with HbA1c <6.5% (43). Another large observational study included 11,812 TBI patients found that greater GV was associated with greater mortality (44). Greater GV after IR were associated with dysregulated energy metabolism, aggravated oxidative stress and exacerbated inflammatory responses, and may lead to poor prognosis (9, 10, 12). In addition, the novel and important effect of insulin signaling on CNS has been widely studied (45). Insulin signaling was participated in neuronal survival and synaptic plasticity (46), while impaired brain insulin signaling was associated with exacerbated neuroinflammation (47), aggravated glutamate excitotoxicity (48), decreased synaptosomal insulin responsiveness (49).

The effects of IR on the prognosis of TBI are mainly due to the impairment of normal insulin action on modulating peripheral metabolism and protecting brain function. It is well studied that insulin signaling cascade promotes glucose and lipid metabolism, protein synthesis, and cell/neuron survival through activation of phosphatidylinositol-3-kinase/Akt pathway (PI3K/Akt) and mitogen-activated protein kinases/Ras pathway (MAPK/Ras) both in the brain and in the periphery (8, 50). IR after TBI leads to dysfunction in neurogenesis, brain function, and whole-body energy balance and metabolism (13).

Therefore, therapy on insulin signaling may be beneficial both in glycemic normalization and neuroprotective effects among patients with TBI (51, 52). Evidences suggested that brain insulin signaling regulates the homeostasis of peripheral energy metabolism through the autonomic nervous system and hypothalamic–pituitary axis (8, 50). The incretins Glucagon-like peptide 1 (GLP-1) and glucose-dependent insulinotropic peptide (GIP), which were suggested to be efficacy in restoring insulin sensitivity (53), have shown potential in reducing GV in patients with Type 2 Diabetes (54, 55). The neuroprotective effect of insulin administration has been reported *in vivo* (56) and in animal models (50, 51, 57) by alleviating glutamate excitotoxicity and reducing inflammatory response. Insulin-like growth factor I (another ligand of insulin receptor signaling) (58, 59), Thiazolidinedione (TZD, known as insulin sensitizing drug) (60, 61), and the incretins GLP-1 and GIP (62, 63) were all reported efficacy in preventing apoptosis, oxidative stress, and neuroinflammation and improving neurological deficit after TBI in animal model. A TZD drug, Pioglitazone, was reported to be associated with a lower risk of recurrent ischemic events in a multicenter, double-blind trial involving non-diabetic patients with IR (64). In addition, several incretin-based therapies have also been approved in Clinical Trials (63). These results make ameliorating IR as a potential approach to TBI treatment, yet which needs concerted efforts to conduct more comprehensive research.

### Limitation

There are several limitations in our study. First, our study is a single-center observational study with relatively strict enrollment. Our study population provided us the opportunity to explore the impact of IR on the prognosis after isolated TBI in a small sample, yet the findings of our study cohort cannot be extended to TBI patients with severe multiple injuries. The small sample size also limits our further analysis on the subgroup that HOMA and FBG are not well correlated. The insight into this subgroup may lead to a more comprehensive perspective on the IR and hyperglycemia after TBI and their effects on the prognosis after TBI. We will focus on this unique subgroup in future studies. Second, because of the applicability of the HOMA2 calculator to glucose and insulin concentrations, four cases were excluded in our study, which may bias the results. In further HOMA studies, multiple sampling to avoid single test error and unusable sample value may be recommended (30). The comprehensive application of over one simple insulin sensitivity index should be considered to better evaluate the insulin sensitivity of the patients. In addition, the impaired insulin sensitivity is not constant (65), and the dynamic assessment of insulin sensitivity and a metabolic follow-up may be more helpful for the TBI treatment. Last but not least, we have only discussed the benefits of insulin sensitization therapy based on our findings, and in follow-up studies, we will design trials to investigate the exact effect of insulin sensitization therapy on TBI.

## Conclusion

IR estimated by HOMA2 was associated with greater GV and an unfavorable outcome after isolated moderate-to-severe TBI among non-diabetic patients. Ameliorating impaired insulin sensitivity may be a potential approach to TBI treatment.

## Data availability statement

The raw data supporting the conclusions of this article will be made available by the authors, without undue reservation.

## Ethics statement

The studies involving human participants were reviewed and approved by the Ethics Committee of Jiangyin Hospital Affiliated to Nantong University. The patients/participants provided their written informed consent to participate in this study.

## Author contributions

CC, HG, and WW were involved in the design of study. CC wrote the manuscript. CC and HW collected all the data and involved in the statistical analysis. HG and WW evaluated the results and revised the manuscript. All authors contributed to the article and approved the submitted version.

## Funding

HG was supported by the Taihu Lake Talent Program (2021) of Wuxi City.

## Conflict of interest

The authors declare that the research was conducted in the absence of any commercial or financial relationships that could be construed as a potential conflict of interest.

## Publisher's note

All claims expressed in this article are solely those of the authors and do not necessarily represent those of their affiliated organizations, or those of the publisher, the editors and the reviewers. Any product that may be evaluated in this article, or claim that may be made by its manufacturer, is not guaranteed or endorsed by the publisher.
